# Performance Analysis of PCM Ceiling Coupling with Earth-Air Heat Exchanger for Building Cooling

**DOI:** 10.3390/ma13132890

**Published:** 2020-06-27

**Authors:** Shilei Lu, Bin Liang, Xinhua Li, Xiangfei Kong, Wei Jia, Lu Wang

**Affiliations:** 1 School of Environmental Science and Engineering, Tianjin University, Tianjin 300350, China; liangbin97@tju.edu.cn (B.L.); lixinhua@tju.edu.cn (X.L.); 2 Tianjin Key Laboratory of Built Environment and Energy Application, Tianjin University, Tianjin 300350, China; 3 School of Energy and Environmental Engineering, Hebei University of Technology, Tianjin 300401, China; xfkong@hebut.edu.cn (X.K.); 201721304001@stu.hebut.edu.cn (L.W.); 4 Beijing Huahou Energy Technology Co., Ltd., Beijing 101500, China; jiawei@huahouet.com

**Keywords:** phase change material, ventilated ceiling, earth-to-air heat exchanger, operation strategy

## Abstract

In recent years, the systematic application of phase change materials (PCM) is continuously developing. In this paper, an innovative PCM ceiling coupled with earth-air heat exchanger (EAHE) cooling system was proposed for building cooling. The system aimed to combine the cooling capacity of soil and the energy storage capacity of PCM, thus improving the indoor thermal environment. Performance of the system was tested by experimental method while data analysis focused on the indoor side. To research the effect of cold storage time on the performance of the system, two different operation strategies were adopted for comparison: 8-h cold storage strategy and 12-h cold storage strategy. Moreover, a control group was set up to observe the performance of the system on indoor temperature under the same weather conditions. The result showed that the experimental room in which we installed this system could reduce peak temperature by 2.1 °C under 8-h timed cold storage strategy and 2.7 °C under 12-h timed cold storage strategy. What is more, under the two operation strategies, temperature and heat flux of the PCM ceiling had similar distribution characteristics. Different strategies mainly affected the sustainability of the system and phase transition efficiency of the PCM ceiling.

## 1. Introduction

Building energy consumption has increased rapidly in the last decade. Building energy consumption accounts for 40% of global energy consumption [1]. Beyond that, air conditioning systems consume about 67% of the building energy [2]. In addition, in China, a large amount of coal combustion has caused severe damage to the atmospheric environment. Under such a circumstance, clean energy and energy storage technology have broad application requirements. 

The usage of PCM to store energy in the form of latent heat is increasing. They make the volume storage capacity of the thermal energy storage (TES) system significantly larger than that of the sensible heat storage system [3]. The thermal performance of different PCM in TES systems is continuously optimized [4,5]. With appropriate design and choices, the phase change temperature of the PCM appeared within the range of indoor thermal comfort, which will definitely permit direct applications of PCM in the building environment [6,7,8,9] and improve building energy efficiency [10,11]. The usage of PCM in buildings could efficiently overcome overheating on hot days, decrease the indoor air temperature [12], and solve the mismatch between users’ load demand and the energy supply [13,14]. Since the 1980s, PCM gradually began to be encapsulated in the envelope structure, accompanied with different kinds of applications [15]. PCM walls were the most common application [16,17,18,19] while PCM windows [20,21,22] and PCM floors [23,24,25] also got extensive research. In addition to the above applications, a number of research indicated that PCM ceilings could also perform effectively to enable peak shaving control [26,27]. In renovation projects or lightweight buildings, traditional thermally active building systems (TABS) are not suitable due to problems such as load-bearing capacity. At this time, the PCM ceiling system with a reasonable price and good energy-saving effect becomes a viable substitute for TABS, and the cost performance of PCM ceiling system is higher under high cooling load [28]. At present, in addition to parametric analysis [29] and efficiency optimization [30], the international research on PCM ceilings has focused more on the innovation of geometry. Alizadeh et al. [31] tested a ceiling fan-assisted ventilation system coupled with PCM. Based on the result, the proposed system has the potential to shift cooling/heating energy demand away from peak hours. Barzin et al. [32] combined night ventilation with PCM-impregnated gypsum board using automatic control technology. As a consequence, when night ventilation was used to charge the PCM, a weekly electricity saving of 73% was achieved. Lin et al. [33] proposed a ceiling ventilation system which composed solar photovoltaic thermal (PVT) collectors with PCM. By using the system in winter conditions, this PVT-PCM integrated ventilation system could improve the indoor thermal comfort of passive buildings efficiently. However, these studies directly used outdoor air or indoor air to help PCM achieve the process of phase transition. In fact, fluctuations in air temperature were uncertain factors. Seasonal changes and unpredictable severe weather were likely to have a greater impact on the stable operation of the system.

As an important utilization form of geothermal energy in the building sector, earth-air heat exchanger (EAHE) system has stupendous development potential. EAHE systems have been widely applied to promote sufficient space cooling [34] and reduce the building energy consumption [35,36,37]. In recent years, a number of research focused on combining EAHE system with other technologies. Among these research, some study on the combination of EAHE system and PCM have achieved great results, mainly focused on the optimization of tunnel cooling capacity [38,39] and the temperature stability of tunnel outlet [40,41]. In the study of Zhou et al. [38,39], simulation and experimental methods were adopted. The result revealed that, with the assistance of PCM, the cooling effect of the EAHE system can be improved effectively. For vertical earth-to-air heat exchanger (VEAHE) system, Liu et al. used tubular [40] and annular [41] PCM modules and realized the decrease of outlet air temperature fluctuation.

Based on the above summary, it was found that both the EAHE system and the PCM ceiling have been researched in many ways, but a combination of these two devices was rarely studied. In addition, while PCM was used as a tool to improve the performance of the EAHE system in previous studies, there was almost no mention of using the EAHE system as an auxiliary tool for PCM systems. If the two can be combined with a reasonable strategy, they will play a role in energy saving and reducing the operating costs. In China, one characteristic policy was high electricity prices during daytime and low electricity prices during the night. Thus, reducing the operating load of air conditioning during daytime has a certain benefit for saving operating costs and energy. More specifically, under the premise of a good air, refreshing effect, if a certain device can provide cold air with stable temperature to help the PCM ceiling finish cool storage at night, the refrigeration module of the air conditioning does not need to operate with the full load during the hot daytime. There is no doubt that the EAHE system is attemptable.

To explore the actual performance of this combination and the method to formulate reasonable operation strategy, innovative PCM ceiling coupled with EAHE system was proposed in this paper, which used soil as the cold source and indoor ceiling as a part of the indoor cooling end. In this paper, the experimental system was manufactured according to the actual size. Other than that, two bungalows were contrasted in this experiment: One operated with this system while the other without. To explore the practical application effects, this research focused on performance of the indoor side and did not conduct an in-depth analysis of the EAHE system. Data collection and analysis were performed under two operating strategies.

## 2. Materials and Methods 

### 2.1. Experimental Area and Climate

The location of the experiment in this study was Tianjin, China. Tianjin is located on the east coast of the Eurasian continent at mid-latitudes and it is the area where the East Asian monsoon prevails. The experiments were all conducted in summer. Main meteorological parameters of Tianjin are shown in Table 1.

### 2.2. System Description 

**Figure 1:**
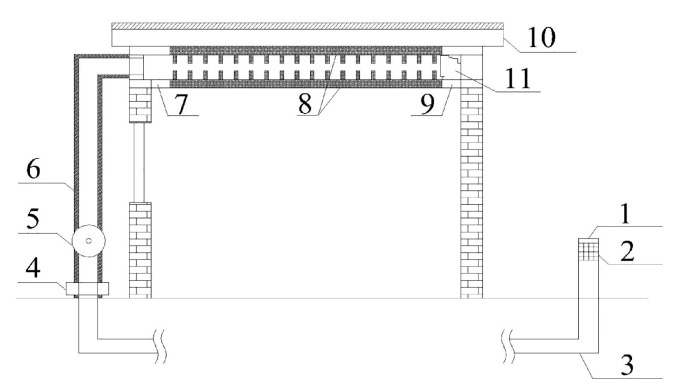
Composition of the experimental room (1-Outdoor air inlet, 2-Air filer, 3-Air duct, 4-Dehumidifier, 5-Duct fan, 6-Insulation, 7-Ceiling fan 2, 8-PCM ceiling, 9-Ceiling fan 1, 10-Roof, 11-Ceiling exit duct).

For the sake of practical experimental results, two rooms with east-west orientation were built in Tianjin, China. The experimental room and the reference room were of the same size, measuring 1.7 m × 1.7 m × 2.1 m. In both rooms, there was a single glass window on the west wall and a colored, steel door on the south wall. As shown in Figure 1, the upper section of the experimental room was equipped with the new type of PCM ceiling. The outlet of the EAHE system was covered by an iron grid with high-density pores, thus distributing the outlet airflow to different directions evenly. Additionally, the gap between PCM ceiling and the surrounding wall was filled with polystyrene board and foam sealant to ensure the airtightness of the ceiling space. For convenience, the experimental room was named as Room 1 while the reference room with a common ceiling was named as Room 2 in following description. 

**Figure 2:**
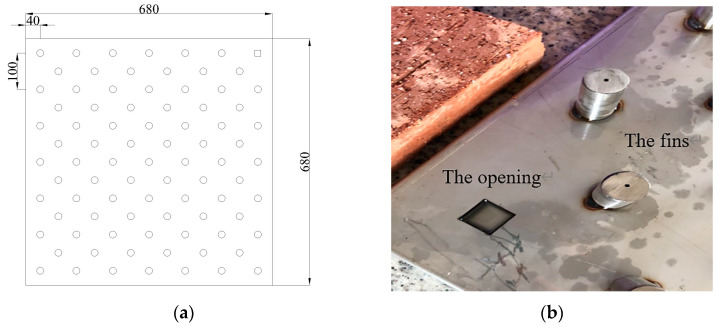
Geometry structure of the PCM plate: (**a**) Structure diagram; (**b**) Real object.

The PCM ceiling was composed of four PCM plates with fins distributed on the surface. Each plate had the same size of 0.68 m × 0.68 m × 0.15 m. A total of 84 cylindrical hollow fins (Radius = 10 mm, Height = 50 mm) was arranged on the surface of each plate, fixed by electric welding. After the fan was started, the air passed PCM plates horizontally from the ceiling entrance under the effect of forced convection. The staggered, cylindrical, hollow fins increased the heat exchange area between air and PCM while strengthening the disturbance to the air flow. Therefore, the effect of convection heat transfer was improved. Additionally, openings were set on the surface of plates which were used for canning PCM and discharging pressure, as seen in Figure 2. Combined with the stereotyped structure, PCM was filled evenly inside the plate for three layers: The surface layer, the middle layer, and the bottom layer, respectively.

In the EAHE system, a single polyvinyl chloride pipe with corrosion resistance was chosen. After the geometric appearance of the tunnel was determined, the EAHE system was tested at five different flow rates. As a result, a speed of 2.5 m/s in the tunnel could provide the most stable outlet temperature. Table 2 shows the main parameters of the EAHE system. According to the pretest result, the average outlet temperature of the EAHE system fluctuated between 20 and 22 °C approximately. Since the ceiling inlet was equivalent to the outlet of the EAHE system, consider choosing the PCM based on the outlet temperature of the EAHE system.

### 2.3. Preparation of the PCM

Aiming to reduce the impact of subcooling as much as possible, organic PCM were selected as fillers. As a kind of PCM which is often used in buildings, higher fatty alcohol has the advantages of low price and large latent heat of phase change. However, multiple crystallization often occurs during its solidification process, which made it not suitable for single use. It has been proven that the combination of higher fatty acid and higher fatty alcohol can weaken the multiple crystallization phenomenon effectively [42]. Therefore, a binary eutectic mixture of higher fatty acid and higher fatty alcohol was used to fill the phase change ceiling.

**Figure 3:**
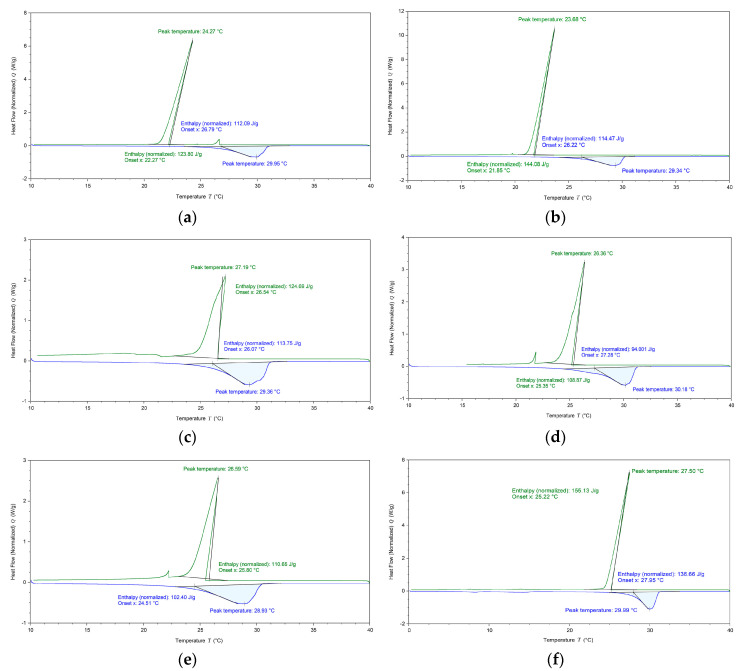
DSC test curves of different DE mass fractions (**a**) 3%; (**b**) 4%; (**c**) 5%; (**d**) 6%; (**e**) 7%; (**f**) 4.87%.

Based on the tested outlet temperature of the underground tunnel, preliminary consideration was given to using n-capric acid (CA) with a solidification temperature of 26.8 °C as the PCM. Still, since its melting temperature was over 31 °C, the heat absorption effect might not be good during daytime. Aiming to reduce the melting temperature, dodecanol (DE) was added to CA at different mass fractions to prepare composite PCM with different melting points, freezing points, and latent heat. It was proven through the previous experiment that 5% DE mixed with 95% CA was suitable for summer cold storage [42]. On this basis, several adjacent ratios were tested by differential scanning calorimetry (DSC). The scanning rate for the DSC curves was 1 °C/min. Curves are shown in Figure 3.

As can be seen, freezing points in Figure 3a,b were 22.27 and 21.85 °C, respectively, which were too close to the outlet temperature of the tunnel. So, these two ratios were not suitable for the experiment. Other than that, absorption peaks of Figure 3c,e were not sharp enough, which will cause a poor solidification effect in practical applications. As shown in Figure 3d, when the mass fraction of DE was 6%, latent heat of melting and latent heat of solidification were significantly lower than other ratios, 94 and 108.9 J/g, respectively. According to a comprehensive comparison, the optimal mass fraction of DE was 4.87%. As seen in Figure 3f, latent heat of melting and solidification were the highest, 136.7 and 155.1 J/g, respectively. Moreover, the absorption peak of the solidification process was sharp enough under this ratio. Table 3 shows the physical parameters of the 4.87% DE-95.13% CA mixture.

To test the durability of 4.87% CA-95.13% DE, constant temperature water was used to circulate it rapidly within the temperature range of 10–50 °C. The melting and solidification process were repeated 1000 times in total. The samples taken from the first cycle, the 500th cycle, and the 1000th cycle were tested by DSC. Table 4 summarizes the test result. It can be found from the table that the phase change parameters of 4.87% CA-95.13% DE changed little during the cycle. After 500 cycles, latent heat of melting process and latent heat of solidification process reduced by 2.8% and 0.3%, respectively. After 1000 cycles, latent heat of melting process and latent heat of solidification process reduced by 4.8% and 0.5%, respectively. The result of a multiple cycle test proved the good durability and stability of 4.87% CA-95.13% DE. Aiming to test the encapsulation effect of 4.87% CA-95.13% DE, a 1000-cycle test was conducted. The test results showed that the leakage rate of the PCM after the 1000th cycles was only 0.79%, which indicated that the encapsulation effect was great.

### 2.4. Operation Strategy

**Figure 4:**
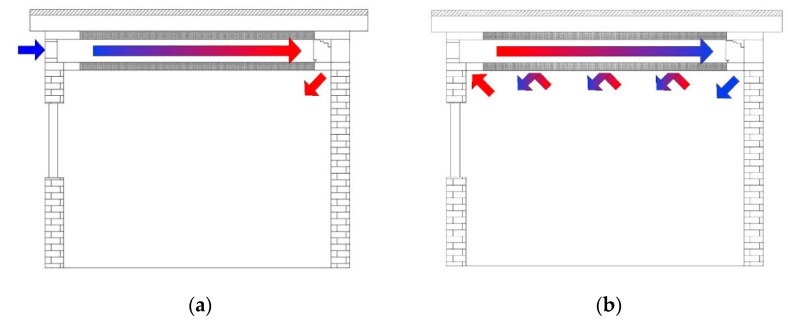
System operation diagram. (**a**) Cold storage process at night; (**b**) cooling process in daytime.

Intermittent operation mode was adopted in the experiment. The EAHE system was opened at night and closed during the daytime. Figure 4 shows the operation diagram of the indoor system. During the night, outdoor air was cooled in the tunnel, passed through the ceiling channel to help the PCM complete the cold storage process. During the daytime, the EAHE system was closed. A part of the uncooled indoor hot air was affected by the density difference, rising continuously to exchange heat with the PCM ceiling. The other part of the indoor air passed through the PCM ceiling with the help of ceiling fan, exchanged heat with solidified PCM, and then was sent to the indoor after being cooled. Through the continuous cycling, the indoor thermal environment would be adjusted gradually.

To find out whether the running time of the EAHE system would affect the performance of the PCM ceiling, two different operation strategies were set up as follows:

Strategy 1: The 8-h timed cold storage during the night (13 July 0:00–16 July 0:00, 72 h in total).

During night, we closed the doors and windows of both rooms. Besides, EAHE system was turned on from 20:00 to the next day 8:00, 12 h in total. The cooled tunnel wind became the cooling source of the room. During this period, PCM stored the cooling capacity while the heated air was sent to indoors, under the action of ceiling fan 1. After that, during the period of 8:00 to 20:00 in the next day, the EAHE system was closed while ceiling fan 1 and ceiling fan 2 were turned on. The PCM ceiling released cooling capacity to indoor air. The cyclic process repeated continuously until the experiment was finished.

Strategy 2: The 12-h timed cold storage during the night (21 July 20:00–24 July 20:00, 72 h in total)

We opened the EAHE system from 22:00 to 6:00 and closed it during the period of 6:00 to 22:00 in the next day. Other parts were the same as Strategy 1.

### 2.5. Measuring Tools

**Figure 5:**
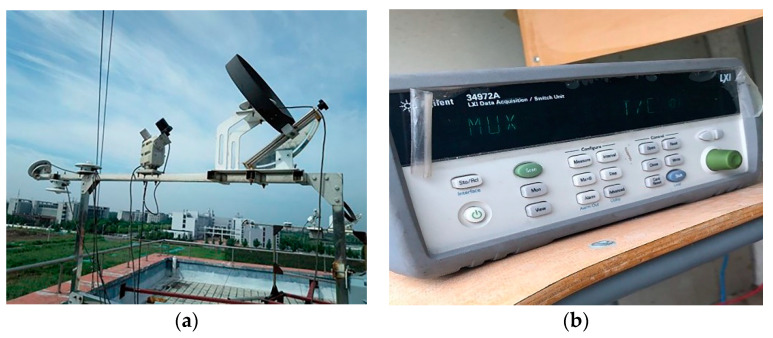
Main test equipment: (**a**) Weather station; (**b**) Data acquisition instrument.

**Figure 6:**
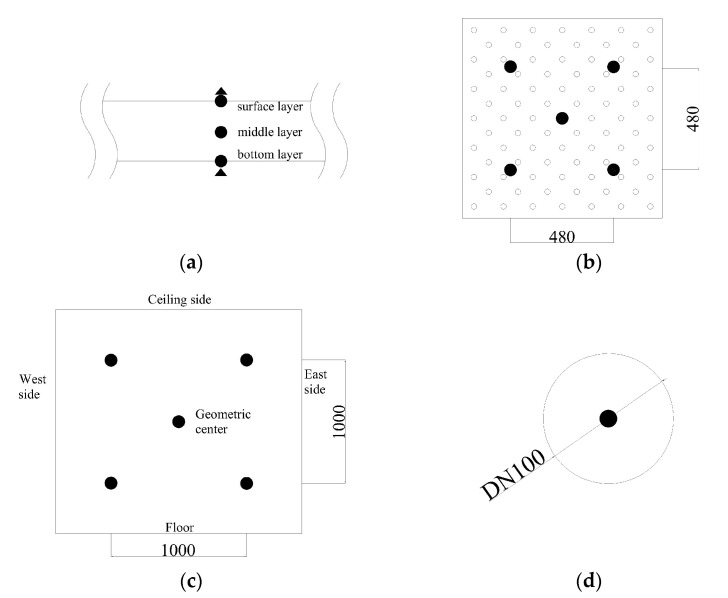
Arrangement of measured points. (**a**) Arrangement of measured points in PCM plate (along the thickness direction), (**b**) arrangement of measured points in PCM plate (middle layer), (**c**) arrangement of measured points in Room 1 and Room 2, (**d**) arrangement of measured point in ceiling inlet.

The experimental test parameters mainly included indoor thermal environment parameters, outdoor meteorological parameters, outlet air temperature of the EAHE system, thermal parameters of the PCM ceiling, and the PCM temperature. The indoor thermal environment parameters included indoor temperature and heat flux, while the outdoor meteorological parameters included outdoor air temperature and solar radiation. As Figure 5a shows, the outdoor meteorological parameters were monitored by a weather station, which was arranged on the ceiling of an energy-saving building on the north side of the experimental room. T-type thermocouple was used to measure all kinds of temperature data. The heat flux sensor was used to measure the heat flux of different ceiling surfaces. Its specification was 15 W/(m^2^·mV). Both the thermocouple and the heat flow sensor were calibrated before the experimental test. All of the temperature and heat flux data were recorded by a data acquisition instrument and synchronized to a USB flash drive, as shown in Figure 5b. All of the data (temperature and heat flux) were measured at one-minute intervals. In the 72-h experiment, all test results were composed of 4320 data points. To sum up, parameters of measuring tools were shown in Table 5. The experimental measurement point layout is shown in Figure 6. It should be noted that the black circles refer to T-type thermocouple while the black triangle refers to heat flux sensor.

## 3. Results and Discussion

### 3.1. Operation Effects of EAHE System under Two Strategies 

**Figure 7:**
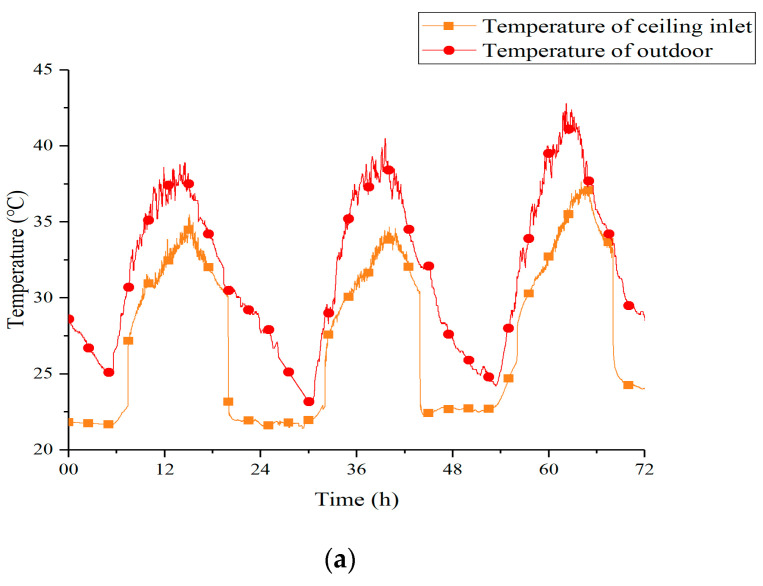

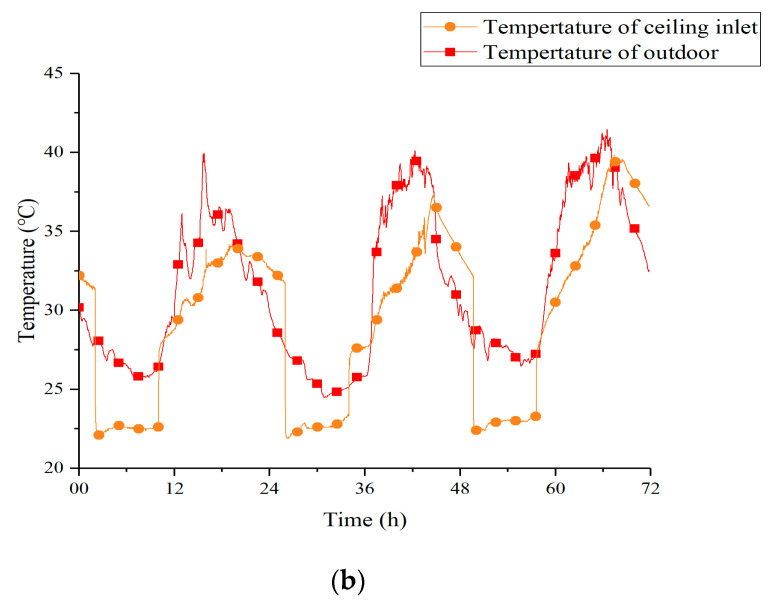
Temperature curves of outdoor and ceiling inlet under two strategies (**a**) 12 h; (**b**) 8 h.

Figure 7 shows temperature curves of outdoor and ceiling inlet under two strategies. Obviously, no matter which strategy was applied, the soil could absorb the heat of outdoor air effectively. As a consequence, a lot of cold air with low temperature fluctuation was provided to the PCM ceiling. Compared with the direct usage of outdoor air, the EAHE system could reduce the temperature fluctuation of cold air by 61% under the Strategy 1 and 84% under the Strategy 2. This result illustrated that the EAHE system could provide more stable air temperature, thus helping the PCM ceiling improve the operating effect.

Table 6 summarizes the cooling effect and the cooling capacity decay of the system under two strategies at night. The cooling effect in the table refers to the decrease of outdoor air temperature after passing through the underground pipes. The two strategies performed similarly in this index. Besides, it could be summarized from the table that whether we chose Strategy 1 or Strategy 2, the cooling effect of the EAHE system would be worse every night, and Strategy 1 behaved more obviously. Thus, reducing the continuous running time per night was conducive to the long-term operation of the system.

### 3.2. Thermal Performance of Room 1 and Room 2 under Two Strategies

**Figure 8:**
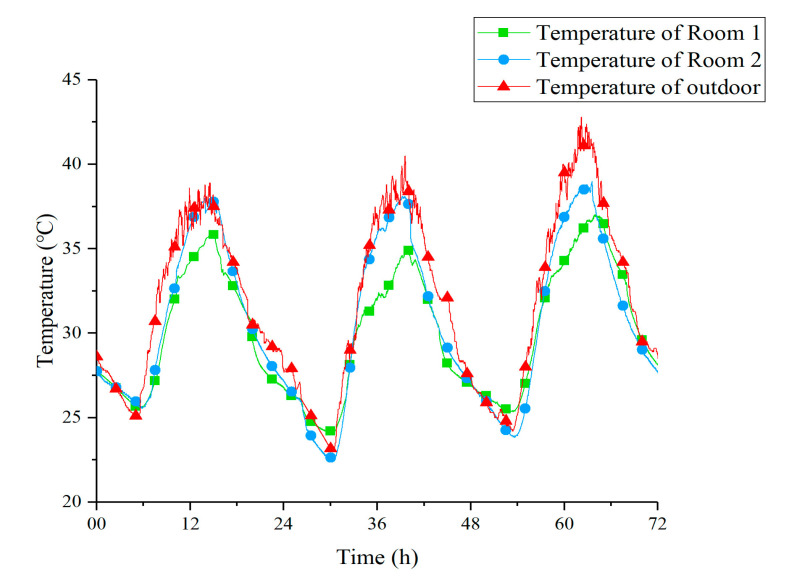
Temperature curves of Room 1, Room 2, and outdoor under Strategy 1.

Figure 8 shows the temperature curves of Room 1, Room 2, and outdoor under Strategy 1. From these curves, it is clear that, compared with outdoor and Room 2, the peak temperature of Room 1 was significantly reduced. This made the indoor temperature more stable over a 24-h period. Table 7 shows the value of three peak temperatures within three days and their relationship. *T*_s_, *T*_d_, and *T*_o_ represent the temperature of Room 1, Room 2, and outdoor, respectively. At night and early morning, the indoor temperature of Room 1 was slightly higher than outdoor and Room 2. It might have been because the doors and windows of the Room 1 were closed while the PCM ceiling released heat to the low-temperature indoor air. On the other hand, the time when the peak temperature appeared in Room 1 was delayed for 126 min compared to Room 2 on average, which indicated that the presence of the PCM ceiling increased the thermal inertia of Room 1.

Obviously, the outdoor temperature on 15 July was significantly higher than the other two days. Due to greater heat exchange temperature difference, the cooling effect on this day should be the best, but it actually did not perform as well as the other two days with the lowest Δ*T*_1_, as shown in Table 7. The possible reason might be just the high-temperature environment outside. Strong radiation and high-temperature outdoor air transferred a large amount of heat to the upper ceiling through the heat conduction of the roof. Under the influence of external disturbance, the cooling effect of the upper PCM ceiling on the indoor air was weakened.

**Figure 9:**
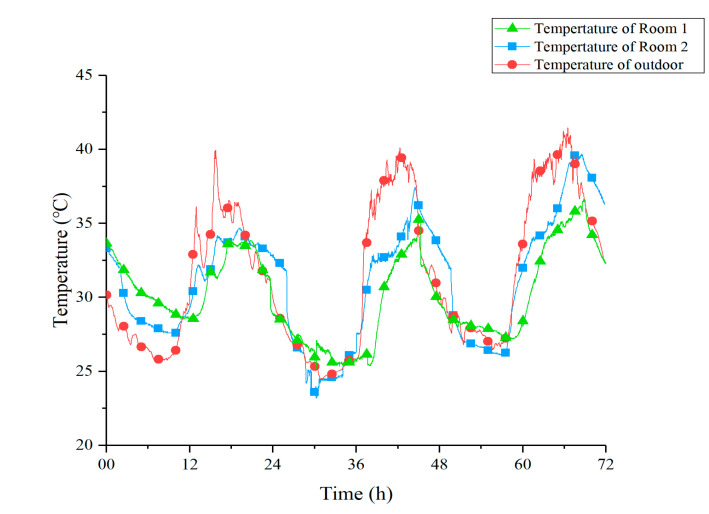
Temperature curves of Room 1, Room 2, and outdoor under Strategy 2.

Figure 9 shows the temperature curves of Room 1, Room 2, and outdoor under Strategy 2. By observing the curves, it is obvious that under the condition of 8-h timed cooling storage, the PCM ceiling could also reduce the peak temperature of Room 1 effectively. Moreover, contrasted with Room 2, the appearance of the peak temperature in Room1 was delayed for 92 min on average. Table 8 shows the data of three peak temperatures within three days and their discrepancy.

A comprehensive comparison of the two strategies is presented in Table 9. Data in this table are the arithmetic mean of temperature index (*T*_s_, *T*_d_, *T*_o_, ∆*T*_1_, ∆*T*_2_) for the entire 72 h. It is worth noting that Strategy 1 brings a better indoor cooling effect. This might be due to a more adequate cold storage time at night.

**Figure 10:**
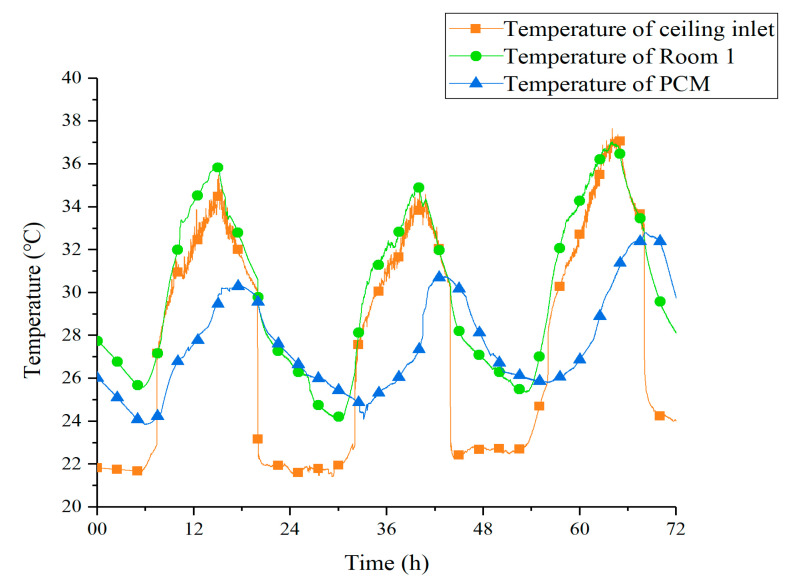
Temperature curves of PCM, tunnel outlet, and Room 1 under Strategy 1.

Figure 10 shows the temperature curves of tunnel outlet, Room 1, and Room 1’s PCM ceiling under Strategy 1. The outlet temperature of the underground tunnel was basically between 21–23 °C with a minor fluctuation. Such stable low-temperature air ensured that the PCM could solidify and melt under sufficient temperature difference. On the evening of the 13th, the latent heat of PCM was released within 9 h at night, from 23:00 to 8:00. Due to the larger temperature difference during the daytime, the melting process was completed in less than 4 h in each experimental day. The fan in the EAHE system was turned off during the daytime, so the temperature at the outlet of the underground tunnel was basically equal to the indoor air, which was absorbed into the ceiling tunnel.

**Figure 11:**
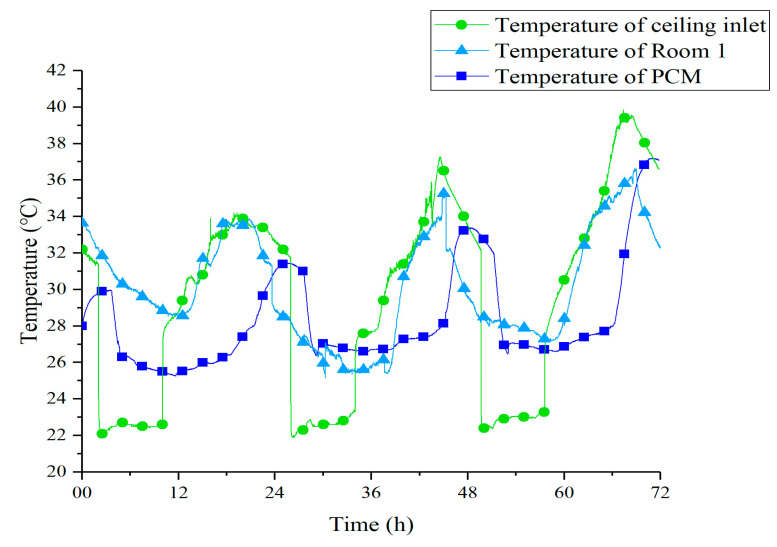
Temperature curves of PCM, tunnel outlet, and Room 2 under Strategy 2.

Figure 11 shows the temperature curves of tunnel outlet, Room 1, and Room 1’s PCM ceiling under Strategy 2. It can be observed that the outdoor temperature shows a growing trend from the 22 July to the 24 July, which led to acceleration of the melting process, measuring 262 min, 197 min, and 133 min. The outlet temperature of the underground tunnel increased about 1 °C compared to Strategy 1. This caused a reduction in ceiling cooling efficiency at night. What is more, under the effect of shorter cold storage time and smaller temperature difference, the PCM ceiling did not achieve a complete solidification process on the 23 July and 24 July. As a result, the peak temperature of PCM on these two days was significantly higher than that of the first day. This phenomenon revealed that, in the case of poor solidification effect at night, the PCM ceiling relied more on sensible heat to reduce the indoor temperature during daytime.

### 3.3. Vertical Contrast of Ceiling Temperature

**Figure 12:**
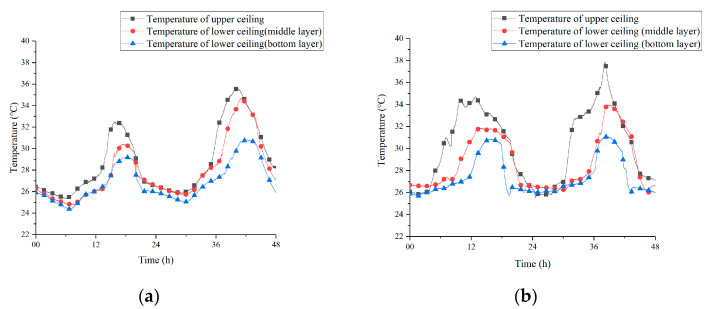
Vertical contrast of ceiling temperature under the two strategies (**a**) 12 h; (**b**) 8 h.

Figure 12 shows the temperature curves of the PCM inside the upper ceiling and the lower ceiling under two strategies. The temperature of PCM at different vertical positions was recorded for 48 h. As a whole, no matter which operating strategy was adopted, the temperature of PCM in the upper ceiling was higher than the lower section. The peak temperature of upper ceiling achieved 35.6 °C in Figure 12a and it even broke through 37 °C in Figure 12b. Such high temperatures appeared because the PCM ceiling on the upper side was affected by the outdoor solar radiation and the high-temperature outdoor air in the daytime. As a consequence, the phase transition process in the upper ceiling became faster and then its temperature increased continuously.

The temperature in the middle layer of the underside ceiling measured lower than that of the upper ceiling and its peak temperature appeared with a delay of 39 and 77 min on average compared to the upper ceiling under two strategies. Additionally, the bottom layer of the lower ceiling showed the lowest temperature among the three. In fact, indoor hot air attached to the indoor surface of the lower ceiling at a negligible speed, which was not conducive to the heat transfer. Maybe the increase of its temperature was mainly due to the heat conduction with PCM in the middle layer.

### 3.4. Horizontal Contrast of Ceiling Temperature

**Figure 13:**
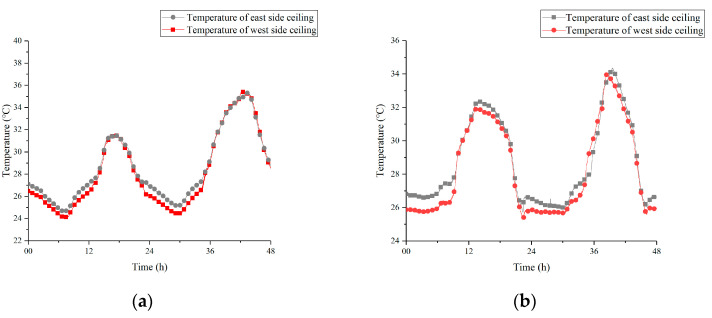
Horizontal contrast of ceiling temperature under two strategies (**a**) 12 h; (**b**) 8 h.

Figure 13 shows the temperature curve of the PCM in the west side of the ceiling and the east side of the ceiling under two strategies. By comparing the two curves in Figure 13a,b, it can be found that the distribution of ceiling temperature had similar characteristics, which mainly reflected in two aspects. On one hand, the temperature of the west side ceiling was lower than that of the east side at night, because the cold air treated by soil entered the ceiling from the west side. On the other hand, the temperature difference between the ceilings on both sides was tiny during the daytime, probably due to a higher heat exchange temperature difference and a higher circulating flow velocity in the daytime.

### 3.5. Comparison of Heat Flux on Different Ceiling Surfaces

**Figure 14:**
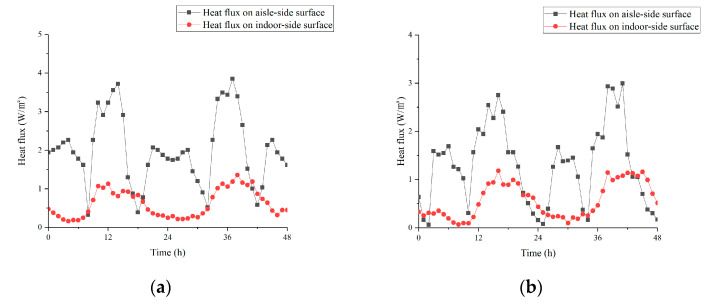
Heat flux of different ceiling surfaces under two strategies (**a**) 12 h; (**b**) 8 h.

Figure 14 shows the heat flux on the indoor-side surface of the ceiling and aisle-side surface under two strategies. To focus on the performance of convective heat transfer between ceiling and air, the radiation part was removed. The test time lasted for 48 h, too. As Figure 14a shows, without enhanced heat transfer measure on the indoor-side surface of the ceiling, indoor hot air adhered to it at a negligible flow rate under the effect of density difference during daytime. The average value of its heat flux was only about 0.9 W/m^2^. At night, the temperature difference between PCM and indoor air was smaller than daytime. The average heat flux decreased to 0.23 W/m^2^.

Observing the heat flux curve on the aisle-side surface of the ceiling, it could be seen that during the daytime, under the operation of the ceiling channel, the heat flux could maintain above 3 W/m^2^ under Strategy 1 while above 2 W/m^2^ under Strategy 2. This result proved that the fins effectively strengthen the convective heat transfer effect between ceiling and air. Since the temperature difference between the tunnel air and the PCM was relatively small, the heat flux of aisle-side was lower than that of the daytime during the operation of the EAHE system at night. However, the low heat flux might also be related to the airflow organization in the ceiling tunnel when the EAHE system was running. This would be the key part of our follow-up research.

### 3.6. Analysis of Phase Transition Duration

**Figure 15:**
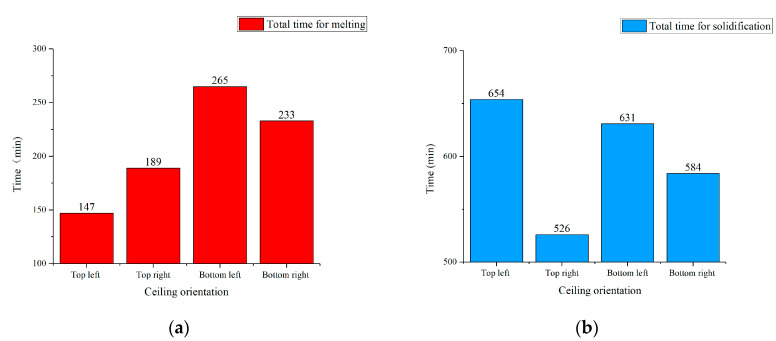
Total time of the phase transition process under Strategy 1: (**a**) Total time for melting; (**b**) Total time for solidification.

In the whole experiment, the solidification process was not complete under Strategy 2. Other than that, regardless of the strategy, it was difficult for the bottom layer of the lower ceiling to fully melt during daytime. So, only the phase transition duration of the middle layer under Strategy 1 was analyzed. Figure 15 shows the total time of the phase transition process under Strategy 1. Throughout the 72-h test (a total of three cycles), the standard deviation of the melting time in Figure 15a did not exceed 4.6, while the standard deviation of the solidification time in Figure 15b did not exceed 7.2. So, data in Figure 15 could reflect the overall performance of the middle layer during experiment days.

From Figure 15a, it can be concluded that the upper ceiling could complete the melting process faster than lower side. The top-left ceiling showed the highest melting efficiency with a total time of 147 min. Despite occupying the closest position to the indoor hot air inlet, the bottom-left ceiling took the longest, 265 min, to fully melt. This result revealed that the PCM in the lower ceiling had a worse melting effect than the upper side. If possible, further optimization on the geometry or heat transfer measures of underside ceiling should be premeditated.

As seen in Figure 15b, the right (east) side of the ceiling took less time to achieve the complete solidification. The top-right ceiling spent the least time while the bottom left ceiling needed above 10 h to finish the process. Other than that, four positions of the ceiling all needed above 8 h (480 min) to finish the full solidification. This result illustrated that Strategy 2 was not enough in cold storage time, indeed.

## 4. Conclusions

The paper described an innovative PCM ceiling coupled with the EAHE cooling system.

The new system used the soil to provide temperature-stable air, thus helping the PCM module to store cold. It was a practical method to overcome the difficulty of using night ventilation strategies in areas with relatively lower climatic cooling potential [43]. In addition, the system only required three fans to run intermittently, whose maximum power was no more than 0.2 kW in total. Besides, optimization for both the PCM ceiling and the EAHE system could improve the performance of the whole system, which makes it have tremendous application potential.

In the research, experiments under two different conditions were carried out for several days. The main conclusions are as follows:

1. Experimental results showed that the 4.87% DE-95.13% CA mixture was suitable for building cooling. However, this combination had the problem that the latent heat of solidification was higher than the one for melting, which was not conducive to the cooling effect of PCM. In this case, it is necessary to further optimize the thermal performance of the composite PCM or find alternatives.
2. For the EAHE system section, excessive continuous running time was not conducive to the sustainability of the earth-to-air heat exchanger.
3. Compared with the room without the newly proposed system, the indoor peak temperature reduced by 2.1 °C under 8-h timed cold storage strategy and 2.7 °C under 12-h timed cold storage strategy with the help of the new system, meaning that the system reduced the indoor peak temperature effectively. Thus, the indoor air conditioning system could undertake less cooling load during daytime.
4. Comparison of multiple sets of data showed that temperature and heat flux of the PCM ceiling had similar distribution characteristics regardless of strategy. Different strategies mainly affected the phase transition efficiency of PCM ceiling. To make fuller use of PCM’s latent heat, enough cold storage time was necessary in practical application.
5. Experimental data proved that ceiling flats with different orientations need different phase transition times while PCM in the bottom layer of the lower ceiling hardly worked. In this case, optimizing the heat exchange effect of the ceiling could start with the air flow organization optimization and the structural adjustment of the ceiling.

## Figures and Tables

**Table 1:** Main meteorological parameters in Tianjin.

Meteorological Parameters	Value
Land surface temperature (°C)	13.5
Land surface temperature amplitude (°C)	15.4
Outdoor dry-bulb temperature (°C)	29.9
Outdoor wind speed (m/s)	2.4
Outdoor air humidity (%)	62.1
Outdoor atmospheric pressure (kPa)	101.7

**Table 2:** Main parameters of the EAHE system.

Parameter	Value
Diameter (mm)	100
Wind velocity (m/s)	2.5
Air flow (m^3^/h)	70.7
Tunnel length (m)	12

**Table 3:** Physical parameters of the 4.87% DE-95.13% CA (30 °C).

Parameter	Value
Thermal conductivity (W/(m·K))	0.16
Specific heat at constant pressure (J/(g·K))	4.82
Density (g/cm^3^)	0.81
Melting range (°C)	27.95–31.02
Melting peak temperature (°C)	29.99
Solidification range (°C)	25.22–27.53
Solidification peak temperature (°C)	27.50

**Table 4:** Phase change parameters of 4.87% CA-95.13% DE in the multiple cycle test.

Number of Cycles	Melting Point (°C)	Freezing Point (°C)	Latent Heat for Solidification Process (J/g)
1th	30.0	27.5	155.1
500th	30.1	27.5	154.6
1000th	30.2	27.4	154.4

**Table 5:** Parameters of measuring tools.

Name	Model	Accuracy
Thermocouple	T-type	± 0.2 °C
Heat flow meter	RLJ-10050C	≤5%
Data logger	34972A	≤0.0041%
Weather station	TINEL-QX5	± 2%

**Table 6:** Outlet temperature performance under two strategies.

Strategy	Cooling Effect (°C)	Cooling Effect Decay between Two Adjacent Nights (°C)
Strategy 1	5.5	0.5
Strategy 2	5.2	0.2

**Table 7:** Peak temperature comparison under Strategy 1.

Parameter	13rd	14th	15th
*T*_s_	35.9	34.9	37.0
*T*_d_	38.8	38.1	39.0
*T*_o_	38.9	40.5	42.7
∆*T*_1_	2.9	3.2	2.0
∆*T*_2_	3.0	5.6	5.7

Note: In the above table, Δ*T*_1_ means *T*_d_ − *T*_s_ and Δ*T*_2_ means *T*_o_ − *T*_s_, °C.

**Table 8:** Peak temperature comparison under Strategy 2.

Parameter	22nd	23rd	24th
*T*_s_	33.9	35.4	36.6
*T*_d_	34.7	37.5	39.9
*T*_o_	40.0	40.1	41.5
∆*T*_1_	0.8	2.1	3.3
∆*T*_2_	6.1	4.7	4.9

Note: In the above table, Δ*T*_1_ means *T*_d_ − *T*_s_ and Δ*T*_2_ means *T*_o_ − *T*_s_, °C.

**Table 9:** Comparison of cooling effect between Strategy 1 and Strategy 2.

Strategy	$\bar{T_{s}}$	$\bar{T_{d}}$	$\bar{T_{0}}$	$\bar{∆T_{1}}$	$\bar{∆T_{2}}$
Strategy 1	35.9	38.6	40.7	2.7	4.8
Strategy 2	35.3	37.4	40.5	2.1	5.2

Note: In the above table, Δ*T*_1_ means *T*_d_ − *T*_s_ and Δ*T*_2_ means *T*_o_ − *T*_s_, °C.
